# American Microscopical Society

**Published:** 1877-03-20

**Authors:** O. G. Mason

**Affiliations:** Secretary


AT THE ANNUAL MEETING of The American Microscopical Society of the City of New York, held Tuesday Evening, January 9th, 1877, the following officers were elected for the ensuing year:

**President*, John B. Rich, M. D., 1 West 38th Street, N. Y.; * *Vice President*, Wm. H. Atkinson, M. D., 41 East 9th Street, N. Y.; *Secretary*, O. G. Mason, Bellevue Hospital, N. Y.; **Treasurer*, T. d'Oremieulx, 7 Winthrop Place, N. Y.; *Curator*, John Frey, Bellevue Hospital, N. Y.

*Re-elected.

O. G. MASON, Secretary.

